# In their own words: a qualitative exploration of patients’ perspectives of genetic counseling sessions across clinical disciplines

**DOI:** 10.1007/s12687-026-00897-5

**Published:** 2026-05-16

**Authors:** Lindsay Peifer, Heather A. Zierhut, Deborah Cragun, T. J. Maresca, Elena R. Fisher, Rebekah Pratt, Krista Redlinger-Grosse

**Affiliations:** 1 Department of Genetics Cell Biology, and Development, University of Minnesota, Minneapolis, MN USA; 2 College of Public Health, University of South Florida, Tampa, FL USA; 3 Department of Family Medicine and Community Health, University of Minnesota, Minneapolis, MN USA; 4 Masonic Cancer Center, University of Minnesota, Minneapolis, MN USA

## Abstract

Genetic counselors (GCs) help individuals navigate living with genetic conditions via models of practice that emphasize GC-defined processes and outcomes. While quantitative, patient-reported measures have assessed patients’ experiences and outcomes, there are limited qualitative studies that explore patient-reported impressions of genetic counseling in their own voice. A subset of genetic counseling patients enrolled in the Genetic Counseling Processes Result in Outcomes (GC-PRO) study completed a semi-structured phone or video interview within 1–2 weeks of their genetic counseling session. Transcripts (*n* = 25) were purposefully sampled from different clinic locations (three institutions), specialties (prenatal, cancer, cardiology, general genetics), participant responses to quantitative surveys, and demographics. A constructivist approach via reflexive thematic analysis identified five themes about the patient experience with GC behaviors and outcomes: (1) Follow my lead; (2) Working collaboratively over time; (3) We value information; (4) GC expertise and caring demeanor are unique; and (5) I left with takeaways. Patients identified the importance of GC behaviors that fostered a patient-led, collaborative session where informational and supportive needs were met in a tailored manner. Clear information-giving from a knowledgeable GC was consistently perceived to be valuable. Participants typically did not enter a session with a predetermined outcome, yet they later identified emotional impacts, gaining new perspectives about genetic information, and feeling empowered by actionable recommendations as outcomes. This research adds to the literature on patient-defined outcomes and perspectives on counseling processes which at times differ from classical provider definitions and serves to support additions to the current models of practice.

## Introduction

Genetic counseling is a growing component of healthcare with the roles of providing medical education, psychosocial support, and discussing familial implications related to inherited medical conditions (Resta et al. 2006). The Reciprocal Engagement Model of Genetic Counseling Practice (REM) emphasizes the importance of the patient and provider influence by characterizing goals for genetic counseling practice and patient outcomes to inform future research and evidence-based practice (Veach et al. 2007; Redlinger-Grosse et al. 2016, Zierhut et al. 2016). Building on the REM, the Framework for Outcomes of Clinical Communication Services (FOCUS) conceptualizes research hypotheses about the impact genetic counseling processes may have on patient outcomes (Cragun and Zierhut 2018). FOCUS also describes and organizes behaviors that genetic counselors (GCs) report engaging in to achieve visit goals and improve those patient outcomes (Zale et al. 2022). While several quantitative patient-reported measures have been developed to broadly assess patients’ experiences and outcomes (Grant et al. 2019; Krousel-Wood 2000; Tercyak et al. 2001; DeMarco et al. 2004; Hibbard et al. 2004, 2005; Barry and Edgman-Levitan 2012; O’Connor 1995; Légaré et al. 2010; McAllister et al. 2011), there remains limited qualitative research that examine patient’s impressions of genetic counselor (GC) behaviors and outcomes in their own voice and across various clinical populations and specialties.

To date, qualitative studies have focused on patient uptake of genetic counseling services or perspectives on genetic counseling from specific patient populations or medical indications. For example, Guimarães et al. (2013) and Crook et al. (2022), who explored the perspectives of individuals diagnosed with or at risk for late-onset neurology conditions, found that patients valued information, emotional support, exploration of family dynamics, and counseling that was personalized to their unique situation (Guimarães et al. 2013; Crook et al. 2022). Garza et al. (2020) sought the perspective of pregnant, immigrant Latinas on various prenatal topics, who expressed appreciation for information and assistance with informed decision-making regarding testing. Barnes et al. (2020) and Rolle et al. (2022) found that the use of inclusive language, counseling environments, and pedigree practices were validating to transgender patients.

Similarly, research on patient perceived outcomes of genetic counseling have been conducted in GC specific populations and/or specialties. A systematic review of qualitative studies by Richardson et al. (2022) found that patients describe many different outcomes from reproductive genetic carrier counseling, including pre- and post-test counseling goals, contextual considerations of further testing and alternative reproductive options, and the perceived impact or utility of the test results. Another study by Semaka and Austin (2019) sought the perspective of patients receiving psychiatric genetic counseling on the process and outcomes of genetic counseling and found that they felt empowered to manage and discuss their mental illness as a result of their GC providing information, personalized support, and space for emotional processing. Within the oncology space, Grant et al. (2006) reported that patients who underwent cancer genetic counseling experienced variable degrees of feeling informed and being satisfied with their testing, while many went on to share information with family members. Pichini et al. (2016) found that adolescents with genetic conditions gained increased personal control of their condition after genetic counseling given an improved understanding of genetics and the ability to anticipate their medical needs and feel in control of their health.

In contrast to the patient- or specialty-specific studies, a qualitative investigation that seeks a broader, and more widely-applicable, understanding of the genetic counseling patient perspective (extending across multiple individual demographics and genetic counseling indications) has not been completed in over 25 years since studies were completed by Bernhardt et al. (2000) and Skirton (2001). These groups found that GC session goals involved education about expectations for the appointment and establishment of a “connection” with the GC. Patients specifically named the thorough length of the session, clear explanation of information, anticipatory guidance, empathy, and ongoing support as facilitators of that connection (Bernhardt et al. 2000). Skirton (2001) focused on the patient perspective in regard to expectations and outcomes of genetic counseling. The main outcome identified was the patients’ “psychological adaptation to the genetic condition in the family” through the genetic professional’s efforts to reduce uncertainty, provide accessible information, and build a connection with the patient (Skirton 2001). The data from the Bernhardt et al. (2000) and Skirton (2001) studies extended across GCs, specialties, and some patient demographics; however, the race and ethnicity identities of participants were not described and, in Bernhardt et al. (2000), some participants were interviewed 2–3 years after their genetic counseling session, potentially reducing recall ability. Additionally, a recent Delphi study indicated that “psychological adaptation” was *not* prioritized as a GC outcome by patients (Redlinger-Grosse et al. 2021). An updated investigation into patient perspectives is warranted to understand whether the findings of these studies have stayed consistent over the past two decades of evolving genomic medicine.

In summary, previous research has identified an accepted model of genetic counseling practice (e.g. REM) and a framework to link this model with patient outcomes (e.g. FOCUS), but incorporation of genetic counseling patients’ perspectives of GC behaviors to inform the foundational elements of practice is limited, especially in regards to a current and broad examination of GC process and outcomes. The Genetic Counseling Processes Result in Outcomes (GC-PRO) study was designed to investigate which GC behaviors are associated with positive patient outcomes amongst diverse racial/ethnic populations across multiple clinical specialties (Fisher et al. 2024). The present study reports on a component of the GC-PRO study that used qualitative interviews conducted shortly after the genetic counseling session to shed light on how patients perceive influential aspects of a genetic counseling session amongst a genetic counseling patient population of diverse patient demographics, clinical specialties, geographical areas, pre- and post-test counseling sessions, and research sites. Specifically, this study sought to explore the research question: what is the patient perspective on GC behaviors and their impact on genetic counseling outcomes? Presently, the models for genetic counseling practice and associated measures of patient outcomes were developed predominantly from the provider perspective. Considerations of current models and frameworks for genetic counseling practice that incorporate up-to-date evidence from both provider *and* patient perspectives can help us more clearly define the diversity of preferred GC behaviors and outcomes. Thus, results of this study may increase understanding of the patient perspective of genetic counseling sessions and ultimately, inform and promote empirically-based and quality genetic counseling care.

## Methodology

### Positionality

The relationship of research team members L.P., H.Z., D.C., K.R.G., and E.R.F. was established prior to the beginning of participant interviews as principal investigators or research members of the GC-PRO study. They all acknowledge their identities as GCs or graduate students in GC programs and this informs their approach to the data given their beliefs in the importance and role of GCs in healthcare and improving patient outcomes. The experiences of the research team as genetic counseling patients was varied and those who have been genetic counseling patients recognize that their experiences also influence how they empathize with and interpret the study patients’ perspectives on GC. One study member, who is not a GC and has not been a genetic counseling patient, is a community-based participatory researcher who works with individuals impacted by genetic risk and disease and thus, is influenced by their perspectives as those impacted by health/genetic risks. Nevertheless, this study was designed and conducted primarily from a provider perspective and thus, participants’ responses may reflect this positionality.

### Participants

Eligible participants included individuals referred for genetic counseling services for themselves or their child due to a hereditary cancer, cardiology, general genetics, or prenatal/reproductive indication. Inclusion criteria consisted of participants who were 18 years or older, English- or Spanish-speaking, and were scheduled to meet with a GC at the M Health Fairview (University of Minnesota), Genome Medical, Inc., or University of South Florida health systems from October 2023 to April 2024.

### Procedures

Eligible individuals were recruited via phone call, email or electronic health system message. All individuals gave informed consent prior to their inclusion in the study. They completed a pre-visit survey, audio recording of their genetic counseling visit, and a post-visit survey as described in Fisher et al. 2024. Patients for the present study underwent genetic counseling with one of 36 participating GCs at the three sites between October 2023 and March 2024. For reference, descriptions of the GCs who conducted the study is described in Fisher et al. 2024. Using quantitative survey data, participants were then purposefully sampled to receive an interview invitation, enriching for individuals with lower scores on survey outcome measures, as well as for diversity of patient demographics, clinical indications, and sites. Participants who were selected to complete the optional follow-up interview were sent an email shortly after their appointment to schedule. Participants met with one of two graduate student researchers (L.P. and T.M.) remotely to complete a 30-minute phone or video call conversation from their home or other private location. Repeat interviews were not carried out and field notes were not taken. The interviewers were trained using a semi-structured interview guide with initial interviews conducted with one of the two principal investigators (H.Z. and D.C.). Participant responses from survey questions were examined prior to interviews to inform prioritization of interview questions for each participant. For example, those individuals who scored lower on an item or measure from the post-visit survey were more likely to be selected for an interview and questioned about the reason for the low score during the interview. Interviews were audio recorded, de-identified, and electronically transcribed. All participants were thanked and compensated for their time as noted in Fisher et al. 2024.

Each interview was completed by one of two graduate student researchers, L.P. or T.M. T.M. joined the research team in November 2023, after interviewing had started. L.P. helped develop the interview guide and T.M. was trained by L.P. to use the guide to conduct interviews. Participants were informed that the interviewers were members of the GC-PRO research team, a study aiming to understand and improve patients’ experiences with genetic counseling.

For the present study, a subset of 25 interview transcripts was selected for analysis. For the first 15 transcripts, five were randomly selected from each of the available University of Minnesota clinical specialties at the time of initial analysis (January 2023): cardiology, oncology, and prenatal/reproductive counseling. The last 10 transcripts were purposefully selected for diversity in responses to survey questions and/or participant demographics with the goal to increase potentially variability in responses or contrasting viewpoints. These final 10 transcripts were selected from all three clinic sites (University of Minnesota, University of South Florida, and Genome Medical) and four clinical specialties (cardiology, oncology, prenatal, and general genetics). Study participation ended after completing the interviews and therefore transcripts, quotations, and results were not returned to the participants for review or comment.

### Instruments/materials

The interview guide consisted of thirteen questions derived from the Genetic Counseling Skills Checklist (GCSC; Hehmeyer et al. 2024) and paired with items from the post-visit survey based on content to help guide interview questioning. The questions covered topics ranging from general patient impressions and satisfaction with the genetic counseling session to questions focusing on specific aspects of the GCSC, such as building rapport, providing education, mutual agenda setting, checking for understanding, facilitating decision making, psychosocial counseling, and promoting patient activation.

### Data analysis

Participant demographics were summarized using descriptive statistics. Qualitative analysis of interview transcripts occurred in NVivo version 14 (QSR International Pty Ltd.). Elucidation of codes, sub-themes, and themes was achieved via a constructivist approach to qualitative research involving reflexive thematic analysis (Braun and Clarke 2022). This inductive approach, in which themes were developed without the limitation of a predetermined theoretical framework, was intentionally chosen to allow the patient voice to inform meaning instead of preconceived notions about what providers have historically identified as important behaviors or outcomes of genetic counseling. Initial codes were created and coding was completed by the graduate student researcher (L.P.). Refinement of codes and determination of themes was an iterative process performed by the graduate student researcher with the guidance of, and in discussion with, student research committee member, K.R.G.

### Human subjects

Approval for this study was obtained from the University of Minnesota’s Single Institutional Review Board (sIRB, Study #00011241), including University of South Florida as a participating site and Genome Medical as a participating research location, in September 2022. The reporting of methodology follows the Reflexive Thematic Analysis Reporting Guidelines (RTARG; Braun and Clarke 2024).

## Analysis

### Demographics

Participant and session demographics are summarized in Table 1. Of the 25 participants, the average age was 38 years and the majority self-identified as female (84%, *n* = 21) and White (64%, *n* = 16). Most participants were adults seeking genetic counseling services for themselves (88%, *n* = 22) and specialties included cardiology (20%, *n* = 5), oncology (24%, *n* = 6), prenatal (44%, *n* = 11), and other/general (12%, *n* = 3). Each participant saw one of 13 GCs mostly for pre-test counseling (92%, *n* = 23) which was conducted through in-person (56%, *n* = 14), video (36%, *n* = 9), or phone (8%, *n* = 2).

**Table 1:** Participant demographics

Variable	Total Participants (*n*=25)	
	Range	Average
Age (years)	21-75	38
	*n*	%
Gender identity		
Female	21	84
Male	3	12
Non-binary	1	4
Race/Ethnicity		
White	16	64
Multiple	3	12
Black/African American	2	8
East Asian	2	8
South Asian	1	4
Hispanic/Latin American	1	4
Patient type		
Adult	22	88
Parent of child	3	12
Specialty		
Prenatal	11	44
Oncology	6	24
Cardiology	5	20
General/other	3	12
Visit Type		
Pre-test	23	92
Post-test	2	8
Visit modality		
In-person	14	56
Video call	9	36
Phone call	2	8

### Reflexive thematic analysis

Five themes reflected patient’s experiences with GC behaviors that impacted their genetic counseling outcomes: (1) Follow my lead; (2) Working collaboratively over time; (3) We value information; (4) GC expertise and caring demeanor are unique; and (5) I left with takeaways.

#### Follow my lead

Participants appreciated the tailored, patient-led nature of their genetic counseling session. It was tailoring that allowed participants to feel that their needs were guiding their time with the GCs and that they were the focus of the GC session. This was specifically noted when GCs used behaviors that allowed for tailoring based on the participants’ informational and supportive needs.

To do this, participants noted that GCs incorporated their specific clinical situation into their informational discussion and recommendations. Sometimes, participants spoke of this in the context of the GC adapting to their level of genetic knowledge or familiarity with the genetic condition. Other times GCs used risk assessment discussions to guide genetic testing recommendations based on specific family history information and patterns. One example is how a GC tailored decision-making support for a patient that had a family history of medical conditions that extended beyond the direct reason for referral.

*I was just happy that [the GC] very much gave me a comprehensive overview of what my options were […] because we were just so unsure of some of the family history and because there were other conditions in my family that I was very pleasantly surprised to hear that I could get comprehensive genetic testing on […]not just the issue I came in with. (Participant 1032)*

When GCs did not attempt to incorporate the patient’s situation into the conversation, participants noticed and indicated damage to rapport. In a family seeking counseling after a diagnosis, they felt that the GC could have looked into their case in more depth prior to the visit for a more personalized, and meaningful, discussion.

*It wasn’t very personable*,* like it wasn’t specific to her condition. And I think that’s made a difference for me because it just felt like she was just seeing another patient*,* that she didn’t really do a deep dive into her specific genetic information…And that’s just*,* I didn’t find it very personal. (Participant 3075)*

While participants spoke prominently on informational tailoring (see subsequent theme), they also spoke about how GCs balanced this information with emotional or decision making support based on their preferences. Participants spoke highly of GCs who seemed to understand what the most important aspects were for each patient and focused the counseling session accordingly.

*…I think I came in for like a specific issue and then we also ended up talking about broader issues. And I think at least currently*,* like I needed the human eye to look at that and to be like*,* you know*,* I see these other issues you might have*,* let’s address those. (Participant 1024)*

One area that was perceived differently by participants was the balance of emotional support and informational support. Specifically, there were some individuals who prioritized more information versus others who wanted more emotional support. Some participants reported requiring more comfort emotionally in order to proceed more confidently and comfortably with the information. GCs who tailored the balance of supportive and information needs of patients created a sense for participants that the session was patient-centered and followed their lead.

#### Working collaboratively over time

Participants praised behaviors from the GC that made them feel individually valued as a patient and an important member of the alliance. This created a sense of collaboration throughout the GC session, as well as in follow-up work. GCs facilitated participant involvement throughout the session with the use of teaching and counseling skills.

Participants consistently praised how and when a GC allowed them opportunities to contribute to the flow of the session by encouraging collaborative, bidirectional exchanges. They appreciated when the GC either left audible space for patient input or verbally invited questions. The conversational flow of the session made them feel at ease and comfortable speaking up, whether explicitly or implicitly invited.

*Having the face-to-face*,* back and forth dialogue helps also make you feel more comfortable and*,* and gives you an opportunity to bring up questions organically where it fits in the conversation. (Participant 1052)*

Participants valued when the GC gave them the opportunity to ask questions and checked-in with them throughout the visit to gauge understanding and modify the discussion accordingly. Some genetic counseling sessions included GC behaviors that intentionally left space for input as well as active questioning about the level of information they were giving. Participants found this helpful in absorbing the information and having all of their concerns addressed.

*She took breaks in between like giving information to kind of let it soak in and see if we had questions. […] There were a lot of little checkpoints along the way. […] She a few times asked*,* “am I giving you too much information? Not enough*,* like right in the middle.” So*,* I think that was helpful too. You could tell she was capable to scale up or down depending on what we*,* what we needed. (Participant 1057)*

Incorporating the participant’s support person was also a GC behavior that felt collaborative and comfortable. Some participants appreciated how the GC changed the level of education they provided when speaking to a partner with a different scientific background. Other participants appreciated their support person being included in the conversation and decision-making process, even stating that it took off pressure from them to speak up or take responsibility for the health condition. This idea was the most prominent in prenatal sessions, where the pregnant person and their partner were encouraged to work through topics of discussion together.

*When it could have all been*,* you know*,* pointed at me because I’m the one with these problems*,* but he also encouraged [my husband] to talk*,* which is good because he’s very quiet. So it didn’t make me feel like I was singled out or a bad mom. (Participant 1056)*

When the GC provided anticipatory guidance on what steps should take place after leaving the genetic counseling visit and the time frame of when follow-up should be expected, participants felt collaborative.

*Being very clear about timelines and like I knew what to expect leaving it*,* I knew where to contact if something did pop up*,* so it just kind of checked off everything that I would hope for in a counseling session. (Participant 1064)*

When this collaborative anticipation was not received from the GC, participants expressed a desire for this through more concrete timelines and action items to be completed. One participant expressed not recognizing this support was needed until sometime after the completion of the genetic counseling visit.

*As a week has passed and I think*,* “Is there something else I’m supposed to be doing to continue to move this whole process forward?” Now I don’t know. So now a week later*,* my answer is*,* “Oh*,* I wish I would have been more clear on what to expect beyond that initial first next step.” (Participant 1044)*.

Participants valued collaboration both during the session, and beyond their direct time with the GC. Some participants highlighted the importance of having faith that the GC will be there for them at every step in the process, including results discussions and navigating management of the health condition after the initial genetic counseling session. Participants felt reassured by having the person that they built a relationship with providing follow-up support.

*Knowing that there’s somebody on the other end of it who’s going to be there also on […] like when I get results and whatnot*,* that it’s going to be the same person who supported me through the decision making in the first place. Who’s going to be the one on the other end of it when I get it back. And that she’s going to be the one to call me and discuss the whole thing with me. (Participant 1001)*

Other participants described feeling comforted when the GC gave them perspective on what to expect in the future regarding potential genetic testing results, impacts of genetic information for family members, natural history of a genetic condition for themselves or their child, and limitations of testing with our current genetic knowledge and technology. This collaborative guidance helped manage patient expectations, assist decision making, and contributed to trust via transparency of clinical processes.

*[I wanted] to know what does it mean that carrying these genes and the result. Not just the result has a negative*,* she explained well about the impact if someone has a positive and carrying the genes what’s going to be the potential issues. (Participant 2010)*

#### We value information

Participants talked about many aspects of information-giving behaviors from the GC that were valued. Upon reflecting on the most helpful or useful part of the genetic counseling session, participants frequently identified desired areas of information that were provided by the GC. The type of information that was valued varied by individual, but participants frequently identified at least one area of education that they remembered being impactful. Identified subject areas included information regarding testing options, the process of testing, what information could be found from genetic testing, and the benefits and limitations of testing. Even participants that had already decided to pursue genetic testing prior to the genetic counseling visit found value in information that validated their decision.

*I didn’t realize that the blood tests that I did also tested for like sex chromosome disorders. I guess that solidified the decision on the first line testing that we did. (Participant 1024)*

Other information valued by participants involved familial risk assessment that highlighted specific risks to other family members, screening recommendations, and which family health risks were more likely genetic.

*A lot of it was just going through my family history*,* just what kind of cancers are there*,* what kind of risks are there? Yeah*,* that was the biggest thing. (Participant 1054)*

Finally, participants appreciated receiving information about relevant genetic conditions, such as a diagnosis or an increased genetic risk, in order to anticipate what to expect with symptomology or management of the condition.

*She went through…[the]definition of Turner Syndrome and then…symptoms that go along with it which I didn’t know that there was any that you could see or I mean*,* feel if you have it. (Participant 1095)*

Overall, participants desired and actively sought information from their genetic counseling sessions and this took precedence over counseling.

*I didn’t talk about [my feelings]. Like*,* I don’t feel the need to talk about my feelings always…but like it doesn’t matter because what we really needed was the information. (Participant 1024)*

Aside from the information itself, participants also described helpful ways GCs presented the information. Participants valued when the GC provided an intuitive flow of information (instead of feeling disjointed) and paid attention to their pacing, avoiding unloading educational points all at once. Information that was understandable also involved simplified terminology and avoiding use of medical jargon.

*I was familiar with the NIPT test before*,* but I thought that she…explained it in a much simpler way than any other person has ever explained it to me. (Participant 1058)*

Some participants acknowledged the GC’s use of visuals during the session. Visuals were either viewed as a nice addition, but not necessary for comprehension, or as a tool that significantly enhanced information comprehension and retention. Individuals in the latter group sometimes described themselves as “visual learners.”

*I couldn’t repeat back everything that is being tested for offhand. I remember the major ones. I remember the “whys” behind various chromosomes. Like I’m a very visual person so to have like the physical diagrams*,* those are stuck in my brain. (Participant 1064)*

Some visual learners that were not provided with diagrams or images during the discussion, described how they wished they had been. One participant stated that a visual may have helped reduce confusion they were experiencing by allowing them to conceptualize the situation and the possible inheritance outcomes.

*I wish I would have had a diagram to say this is what’s normal*,* this is what a lot of people see and this is where I am. And then from there I would have liked to say*,* these are the possible scenarios that could happen. Like this is best case scenario*,* this is worst case scenario. And then maybe circle these are the two situations or three situations that I’m looking at. (Participant 3046)*

Participants also commented on the amount of information that was provided. A few participants praised how the GC presented relevant information in a succinct manner. Some also saw the value of how the GC’s educational process was concise, yet also detailed. Many preferred the thorough education that GCs provided, stating that it helped them feel more informed after the visit and able to communicate what they learned with others.

*I have a record of the things that she was talking about. And so I’m looking at them now and just seeing how thorough they are. I could definitely explain to friends what’s going on and what it looks for and even [that] there are 36 genes that they’re going to look for for colon stuff*,* it’s very specific and in depth and I love that. (Participant 1054)*

Regardless of the participants’ perspective on the thoroughness of information, nearly all participants denied that there was information that should have been removed or shortened. They described all of the information they received as important.

#### GC expertise and caring demeanor are unique

Participants acknowledged that GCs’ blend of genetic expertise and empathy contributed to their educational and supportive behaviors being perceived as unique to other health professionals. Specifically, participants spoke unprompted about the GCs’ trait of knowledgeability, specifically in relationship to the GCs experience and confidence in communicating their knowledge in a tailored way that contributed to them perceiving the GCs as having unique expertise. Others elaborated on this idea in the context of their connection with the GC by explaining that the demonstration of expertise created trust and made them feel more reassured.

*You don’t want someone who doesn’t sound like they know what they’re talking about*,* or hasn’t had these conversations before. She was very confident*,* which is good. (Participant 1010)*

Tailored facilitation of testing decisions were also a part of the expertise that patients appreciated. Participants felt confident in moving forward with testing when GCs explained the benefits of testing and specific reasoning for their recommendations specifically based on the patient’s clinical situation.

*I just think having a better understanding of why they wanted to do the test in my specific case made me feel a bit better because I kind of went in understanding broadly why they would want the test*,* but she was able to give me the specific reasons and that made me feel better. (Participant 1090)*

Certain individuals valued the GC’s expertise over their own ideas, stating that they wanted to do what was best for their health or their child’s health and relied on options presented by the GC’s.

In addition, participants consistently identified a caring, empathic demeanor in their GC as a factor that they liked about their visit, often stating that it made them feel more relaxed and comfortable throughout the conversation. The degree to which they appreciated that aspect, however, differed amongst individuals. Some participants perceived the GC’s demeanor as a crucial component to their positive experience, while others noticed this behavior, yet did not discuss it as a necessity for them. One participant highlighted the GC’s empathic nature as the highlight of their experience and elaborated on its calming effects.

*She just had a really kind demeanor about her*,* so… Something very relaxing having the conversation*,* you know*,* difficult conversation with a perfect stranger when they come across as being kind and genuine…and she really did. (Participant 1052)*

Other participants still appreciated the kindness of the GC, but emphasized their professional demeanor or distance as similarly important in contributing to their expertise. They did not need an emotional invitation to feel more comfortable in the discussion. One participant described this as not desiring an “emotional attachment” with their healthcare provider.

*I don’t look for…an emotional attachment; maybe I see it*,* as in emotion*,* as a different way. But I felt like she was there. She showed empathy and care for me*,* but it was on a professional basis. (Participant 3073)*

Whether patients prefer the kindness or the professionalism of the GC, patients noticed the demeanor of the GC and it contributed to their feelings of being supported.

The time in which the GCs allowed for participants contributed to the way in which GCs expertise and demeanor met their needs. Participants expressed that they entered their genetic counseling sessions with questions and/or that questions arose during the discussion.

*I always care about providers who feel caring and understanding and empathetic. And that’s the feeling that I got*,* just like the patience and the demeanor. Like*,* a lot of times that’s more important to me even than the information ‘cause I felt comfortable that if I didn’t understand*,* I could just ask without her prompting me. (Participant 1054)*

Participants were satisfied with the time spent with the GC when all of their questions were answered. They determined that the session was the right length of time when their concerns were fully addressed, yet not overly drawn out.

*I did feel like it was a very good length of time. Long enough where I was able to get all the information I needed*,* but not*,* I guess*,* drawn out to the point where I was confused by the end of it. (Participant 1095)*

When there was no perceived rush to finish the session, this was an indication to participants that the GC truly cared about them. They felt that time was given in a way that was supportive of their needs, instead of feeling managed or rushed to suit the provider’s other priorities.

*Because we all know that in today’s healthcare world that you get your 15 min and that’s that. And sometimes that’s just really not enough. And so I think when you’re discussing something that could change someone’s life*,* for better or for worse*,* that it’s better to take the time to make sure that you do it right. (Participant 1001)*

#### I left with takeaways

When reflecting on their genetic counseling visit, participants identified key takeways that they took from their sessions. Notably, these takeaways could be interpreted as genetic counseling outcomes or goals even though this language/term was not used by patients. Often, these takeaways were not predetermined, indicating that they didn’t necessarily come to genetic counseling with a desired goal in mind. Yet, participants spoke about outcomes in a reflective nature as prominent takeaways that made them change their thought processes (e.g., how they thought about their genetic risk or condition) or actions they could take to manage their health. Overall, there was not one singular outcome that all participants hoped to achieve from their genetic counseling visit. Rather, individuals named several takeaways of genetic counseling that were impactful including changes in emotional state, a change in perspective based on the genetic information provided, or feeling empowered.

Multiple participants described a change in emotional state as a result of the discussions that took place during the genetic counseling session. Emotional impacts were an identified outcome that could either lead to an increase or decrease in anxiety according to participants. Thus, not all participants described the same change in emotions. Most individuals expressed a feeling of reassurance and calmness from the topics addressed compared to the anxiety that they were feeling prior to the visit. The emotional impact was so pervasive with some participants that it was the prevailing takeaway.

*I was a little anxious going into it*,* partially not knowing what to expect in the appointment at all*,* partially because those things can be scary*,* and honestly it helped calm a lot of that. (Participant 1064)*

This emotional de-escalation and, thus, change in emotional patterns was not felt by all participants. Conversely, one participant described becoming more anxious after discussing risks with the GC. This participant had previously anticipated a lower risk to their family than was expressed by the GC.

*I guess it just made me a little bit nervous*,* thinking*,* […]I was of a higher risk to give my child the disorder that I was born with. And that really hadn’t crossed my mind because mine was not genetic. (Participant 1056)*

Participants also identified informational discussions that re-framed their previous perspective on a myriad of genetic topics. The most frequent opportunities for new insights came from risk assessment discussions. Patients noted that an outcome of genetic counseling was a change in how they viewed the genetic implications of their personal or family clinical situation. One participant expressed how the GC encouraged her to consider the cancer risks for her son when she had only been concerned about her daughter’s risk for the familial genetic condition.

*She said that a lot of these genetic issues can affect males as well as females. And I was like*,* woah. She kind of just like slapped me back into reality a lil' bit because I tend to worry more about my daughter just because she’s a lot like me. (Participant 1001)*

And finally, discussions with the GC frequently gave participants information or recommendations that called them to manage their healthcare differently and in a more empowered way that led to behavioral activation. GCs encouraged them to take actionable steps to promote the health of themselves and their family, especially in the context of familial risk assessment. Some participants were more encouraged to share the information they learned with family, whether that be to pursue genetic counseling or seek out preventative screening measures. Participants found this information useful; it highlighted steps they could take to help themselves and others. One participant received valuable family risk assessment information and screening recommendations of which they were previously unaware.

*She recommended that I*,* and my sister*,* and my mom get cardiac echoes*,* and that was not a recommendation that had been made to me before. […] So that was actually a really important intervention point” (Participant 1109).*

Actionable intervention recommendations from the GC were a valued takeaways or outcomes by participants, resulting in greater feelings of control and proactivity.

## Discussion

This study aimed to better understand the patient’s perspective on genetic counseling behaviors and outcomes and to explore, in the patient’s own words, their experiences with genetic counseling. Five themes were developed from the GC-PRO patient participants, who represent perspectives from diverse backgrounds, specialty areas, and clinics: (1) Follow my lead; (2) Working collaboratively over time; (3) We value information; (4) GC expertise and caring demeanor are unique; and (5) I left with takeaways. When examining these themes, they broadly speak to patients who experienced GC behaviors and outcomes that were tailored given the patient’s varied informational and support needs, the degree of desired collaboration, and the takeaways from genetic counseling that were not predetermined, but rather evolved throughout the session.

Genetic counseling may benefit from establishing a working definition of tailoring or personalization similar to other medical disciples, which typically define tailoring as including the presence of adaptations to language and the use of educational tools (Harrington and Noar 2012; Yu et al. 2022). Zale et al. (2022) assessed the GC perspective of tailoring and the reported tailoring behaviors that participants practice in their genetic counseling sessions. Many of the behaviors described by Zale et al. (2022) are comparable to patient perspectives of tailoring found in this study, such as frequent check-ins during the session, opportunities to interrupt with questions, and testing recommendations based on the patient’s unique clinical situation. It is not clear from the data whether patients experienced, or noticed, the other tailoring behaviors that GCs described in Zale et al. 2022; such as modification of the session based on patient needs, concerns, and expectations, although the way in which patient’s described the GCs following their lead alludes to such modification. Results of this study appear to indicate that patients experience GC behaviors that contributed to tailoring in several ways - their engagement, informational and support needs, and ultimately, genetic counseling takeaways or outcomes.

### Tailored engagement

Participants in this study commended their GC’s effort to tailor and have a collaborative discussion that was bidirectional and relevant to them, especially based on their questions and unique personal and family histories. Patients found that the GC followed their lead in this tailoring and this allowed them to be collaborators in the GC session in an engaged manner. Similarly, research from the nursing and psychiatric fields have found greater patient engagement and activation with communication that was personalized to patients’ attitudes toward managing their health needs (Ruiter et al. 2006; Pellet et al. 2024).

Our results suggest that genetic counseling patients preferred a counselor to assist with continuity of care through their healthcare journey and thus, they found the experience of collaborative care over time to also be valuable. Bernhardt et al. 2000 also found that anticipatory guidance, quality time, and continuity of care were valued by patients; patients appreciated the ability to contact their GC with questions at any point in time. Further elucidation of the benefits of anticipatory guidance and continuity of care given by GCs could describe the role of the GC in the patient care journey as it extends before and after the session.

Coordination of care was also noted by GCs to be a key area of tailoring that patients identified as important. Further investigation into the patient experiences and preferences regarding GC roles in coordination of care is needed to better characterize this preference and implications for the GC-patient relationship over time.

### Tailored information

Information was a highly valued component across all of the genetic counseling sessions as seen through the participants’ praise of the GCs’ information-giving skills and outcomes involving new knowledge and perspectives gained from information. Previous studies have found poorer patient outcomes, including confusion and low compliance with recommendations, when information is not provided at a level they can understand (Mayeaux et al. 1996; Shaw et al. 2009). Accessible educational points were often cited as the most useful part of the genetic counseling visit or something patients were hoping to get from the visit, aligning with prior literature (Bernhardt et al. 2000; Skirton 2001). These ideas are currently supported by the REM tenet, “Genetic information is Key,” as patients are presumed in this model to desire genetic information and find it powerful. Our results also support the process goals of this tenet and the importance of providing pertinent information in a way that is understandable. An outcome described in the REM study and identified in this study involves patients gaining new perspectives as a result of receiving information from their GC (Veach et al. 2007).

The participants in this study actively sought information and accepted the information provided. Previous work has shown that individuals receiving genetic education can differ in the amount of information that they prefer (Mighton et al. 2019). Individual preferences are likely defined by their coping style, education level, and perceived severity of the health condition (Plamann et al. 2021). This indicates that our study population may be enriched with “information seekers,” or “high monitors,” who desire more education. While we targeted the entire population of individuals seeking genetic counseling services in the GC-PRO study, information-motivated individuals may have been more likely to consent to a scientific study and follow-up interview. More research, therefore, is needed to better characterize informational preferences in genetic counseling patient populations widely and in comparison to other medical specialties.

### Tailored support

Study participants varied in how they perceived support from the GCs. While the definition of genetic counseling includes psychosocial support (Resta et al. 2006), forms of support were described by participants as anything from empathy and emotional support to the general kind and professional demeanor of the GC with no specific desire for a deeper connection. This variation may relate to the GC-patient working alliance and the contributing parts of this alliance. Bordin (1979), in describing the therapeutic alliance in psychotherapy, discusses three components of this alliance to include bonds (positive attachments between the counselor and client), tasks (behaviors within the therapy session), and goals (a client’s desired outcome of the session). Thus, the perceived support from the GCs in this study may be an illustration of the tailoring to various aspects of the therapeutic alliance, whether it be relational, behavioral or the outcomes or goals of the session.

Besides initial anxiety over the content to be discussed, participants did not usually report emotionally charged experiences that held a significant place in discussion during their genetic counseling session. A counseling-focused model of genetic counseling would be hypothesized to be very beneficial to some patients but may be unnecessary or even not preferred by others (Austin et al. 2014; Biesecker et al. 2017; Schaa and Biesecker 2024). This may mean that support involves an assessment and understanding of the patient’s informational *and* emotional needs and working to meet these needs could be more effective in practice than specifying a standard education versus counseling educational approach (Roter et al. 2006). GCs approach to genetic counseling should not be either-or; rather, GCs need to ask patients about their needs for education and support in both areas in order to adequately meet them. It is not known from these participants’ responses alone to what extent the GC tailored their support approach to these different patient preference types.

### Tailored outcomes

GCs typically have clear goals for a session, and researchers often measure patient outcomes with well-defined terms, like “activation” and “empowerment” (Krousel-Wood 2000; Hibbard et al. 2004, 2005; Barry and Edgman-Levitan 2012; O’Connor 1995; Légaré et al. 2010; McAllister et al. 2011). Interestingly, participants in this study spoke about “takeaways” form their visit in referencing what professionally is termed “outcomes. Also of note, participantsoften did not have a concrete idea of their preferred takeaways or outcome(s) prior to a session, and/or they left their visit with takeaways or outcomes that they were not expecting. Previous studies have also noted patients’ lack of expectations and awareness of what they can get from genetic counseling since they were often not familiar with the profession (Bernhardt et al. 2000; Skirton 2001). This adds to the prior research that has demonstrated that patients may value different experiences and outcomes than GCs and researchers (Redlinger-Grosse et al. 2021, Zierhut et al. 2016). Conceptualizing outcomes from the researcher or GC perspective assumes incorporation of patient goals, but our data indicates that patients may not have set goals related to outcomes of the session but do leave the genetic counseling session with takeaways; furthermore, conceptualization of these outcomes may arise and then change over the length of the session or even after the session. Therefore, understanding patient-identified goals and connecting them with associated or takeaways that are of importance to the patient remains a challenge for the field. Similarly, the wide variety of takeaways that were reported to be valued by different patients provides some explanation for why finding standardized tools for measuring genetic counseling outcomes or one sole outcome from genetic counseling is difficult.

### Study limitations

Due to the nature of the study design, the participant pool may have been enriched for patients who were actively interested in information, were more likely to seek genetic counseling services, agree to participate in research, and were dedicated to providing their opinions for a follow-up interview. The development of themes focused on the value of information and the educational or anticipatory types of support may have been prominent due to these potential sample characteristics. Patients experiencing more sensitive clinical situations may have been less likely to consent to have their genetic counseling session recorded, potentially leading to less of an emphasis on the importance of emotional support in our consented sample. Similar feelings of anxiety could be enough to prevent non-information seekers from accepting genetic counseling referrals and attending genetic counseling appointments or even from consenting to be part of this study (Mellor 1992; McVay et al. 2013; Reed et al. 2023). Additionally, the genetic counseling sessions in this sample were most often pre-test counseling sessions. Many participants reported not having emotional needs at the time of the pre-test conversation, but they could foresee the importance of that psychosocial counseling piece depending on the results they receive and appreciated that the GC will be there for them at results disclosure. Support and informational needs may be different in unrecorded sessions or a sample enriched for more post-test counseling.

Second, while one semi-structured interview guide was used consistently for all of the participant interviews, edits to question wording and the addition of prompts took place as interviews progressed over the year-long recruitment time period for this present study. The number of questions asked of participants and the follow-up prompting that was utilized varied based on their responses and was limited to a 30-minute interview. Adaptations to the interview could have influenced the types of topics covered during the interviews and how in-depth some content areas were explored, limiting diversity of perspectives within certain themes.

Finally, many elements of diversity and intersectionality of identities were not included in this analysis. Participants were intentionally selected for variability in responses and demographics (e.g. gender, race, and ethnicity) yet these identities may not have been the major contributors to responses. Additionally, identities of the GCs who conducted the participants session may have influenced responses and themes that developed in this study.

### Practice recommendations

Results from the present study support several aspects of the model of genetic counseling practice and outcomes - the REM and FOCUS, respectively (Cragun and Zierhut 2018; Veach et al. 2007). Specifically, patients in this study spoke to GC behaviors that align and contribute to the REM’s tenets of education, patient’s individual attributes and the GC- patient relationship (Veach et al. 2007). Similarly several of the outcomes outlined in FOCUS framework were valued by the patient in regards to GC behaviors that allowed them to leave the GC session with takeaways regarding follow-up testing, management, and care. Unique in this study, however, was the overarching finding that these models cannot be viewed as static; rather there are key GC behaviors that facilitate a nuanced way GCs contribute to the dynamic nature of genetic counseling. From the perspective of the patient, the following are practice recommendations:

- Assessment is critical - The tailoring of patient education and support for the patient starts with GC behaviors that support assessment. GCs cannot tailor discussions adequately without assessing the patient’s needs and desires. This is even more critical given the finding that patients don’t necessarily come to a session with specific goals and outcomes. While the REM and FOCUS do not specifically name or emphasize this dynamic process, it is a critical aspect woven throughout genetic counseling that deserves recognition.
- Tailoring to the patient’s needs - As patients have named in this study, there is variation in regards to their informational needs and desire for emotional support. Some patients value information and thus, warrant education. Others have more psychosocial needs and thus, value more emotional support. It cannot be one size fits all and GCs are nimble in how their behaviors contribute to the tailoring process.
- Outcomes are variable - Similarly, patients brought or established varied takeaways or goals for the GC. As FOCUS demonstrates, there is then a wide-range of genetic counseling outcomes. As noted earlier, this then demonstrates why measurement of genetic counseling outcomes is challenging as a wide-net is needed to capture the variety of outcomes that patients value.

The flexibility required by GCs in assessment and tailoring behaviors to then contribute to the outcomes of genetic counseling deserves acknowledgement and perhaps, warrants revisiting the REM and FOCUS to more fully capture the dynamic nature of this practice.

### Conclusion

Genetic counseling patients offer important perspectives on genetic counseling behaviors and outcomes that can help inform current practice. Patients identified the importance of GC behaviors that fostered a patient-led, collaborative session where their informational and support needs were met in a tailored manner and this allowed them to leave with takeaways. Given the importance of tailoring for patients, this means that patients have variable views on what a successful genetic counseling visit means for them, both in process and outcomes. This suggests an adaptation to the genetic counseling models, and frameworks to include more patient-identified elements, priorities, and recommendations.

## Data Availability

The data that support the findings of this study may be available on request from the corresponding author. The data are not publicly available due to privacy or ethical restrictions.
